# The Biological Properties and Potential Interacting Proteins of d-Alanyl-d-alanine Ligase A from *Mycobacterium tuberculosis*

**DOI:** 10.3390/molecules23020324

**Published:** 2018-02-03

**Authors:** Shufeng Yang, Yuefei Xu, Yan Wang, Feng Ren, Sheng Li, Wenyong Ding, Yufang Ma, Wenli Zhang

**Affiliations:** Department of Biochemistry and Molecular Biology, Dalian Medical University, Dalian 116044, China; shufengyang78@163.com (S.Y.); xuyf0051@163.com (Y.X.); yanwang_2018@163.com (Y.W.); renfeng16@163.com (F.R.); lisheng_1996@163.com (S.L.); dingwy@dlmedu.edu.cn (W.D.)

**Keywords:** *Mycobacterium*, d-alanine-d-alanine ligase, peptidoglycan

## Abstract

(1) Background: d-alanine-d-alanine ligase (DdlA), an effective target for drug development to combat against *Mycobacterium tuberculosis* (*Mtb*), which threatens human health globally, supplies a substrate of d-alanyl-d-alanine for peptidoglycan crosslinking by catalyzing the dimerization of two d-alanines. To obtain a better understanding of DdlA profiles and develop a colorimetric assay for high-throughput inhibitor screening, we focused on explicating and characterizing *Tb*-DdlA. (2) Methods and Results: *Rv2981c* (*ddlA*) was expressed in *Escherichia coli*, and the purified *Tb*-DdlA was identified using (anti)-polyhistidine antibody followed by DdlA activity confirmation by measuring the released orthophosphate via colorimetric assay and the yielded d-alanyl-d-alanine through high performance thin layer chromatography (HP-TLC). The kinetic assays on *Tb*-DdlA indicated that *Tb*-DdlA exhibited a higher affinity to ATP (K_mATP_: 50.327 ± 4.652 μmol/L) than alanine (K_mAla_: 1.011 ± 0.094 mmol/L). A colorimetric assay for *Tb*-DdlA activity was developed for high-throughput screening of DdlA inhibitors in this study. In addition, we presented an analysis on *Tb*-DdlA interaction partners by pull-down assay and MS/MS. Eight putative interaction partners of *Tb*-DdlA were identified. (3) Conclusions: Our dataset provided a valuable resource for exploring *Tb*-DdlA biology, and developed an easy colorimetric assay for screening of *Tb*-DdlA inhibitors.

## 1. Introduction

According to the 2017 statistic report by the WHO [1], Tuberculosis (TB) killed approximately 1.4 million people worldwide in 2016 and an estimated 10.4 million new TB cases occurred annually. The current anti-TB treatment regimens, in particular, remain very unsatisfactory for infection with *Mycobacterium tuberculosis* (*Mtb*) strains of MDR (multidrug-resistance) or XDR (extensively drug-resistance) [1]. *Mtb,* a causative pathogen of TB [1,2,3], has been found to be the dominant strain with MDR or XDR at present. So far, intensive worldwide efforts have been made to discover novel anti-TB drug targets or innovative anti-TB drugs to combat against these evolved *Mtb* strains.

**Figure 1:**
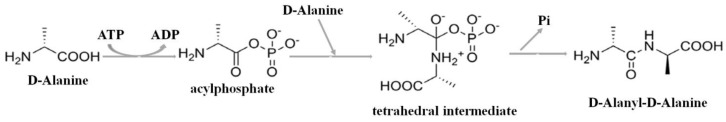
A series of chemical reactions catalyzed by DdlA.

The DdlA (d-alanyl-d-alanine ligase A) in *Mtb*, catalyzing the dimerization of two d-alanine molecules, has been of particular interest as a potential and attractive anti-TB drug target [4,5,6]. The ATP-dependent dimerization reactions catalyzed by DdlA proceed through the phosphorylation of the d-alanine at the carboxyl group by the γ-phosphate of ATP to produce an acylphosphate intermediate firstly. Then, a tetrahedral intermediate is formed by the nucleophilic attacking of the amino nitrogen of the second d-alanine on the acylphosphate intermediate. Finally, a phosphate group is released from the tetrahedral intermediate to generate the final product of d-alanyl-d-alanine (Figure 1) [7,8,9].

d-alanyl-d-alanine is involved in the pentapeptide biosynthesis of peptidoglycan (PG) as a dipeptide donor. PG is a universal and pivotal component of the mycobacterial cell wall. It is fundamental for bacteria to maintain the defined cell morphology, withstand the internal osmotic pressure, and affect cell division [10,11,12,13]. The biosynthesis of PG is an ideal target for anti-TB drug design because the whole pathways are not present in mammalian cells [9]. DdlA is one of the potent candidates as an anti-TB drug target among a great deal of factors that affect PG biosynthesis because (1) it utilizes d-alanine as its substrate, which is specific for bacterial PG biosynthesis; (2) it is essential in mycobacteria, and its essentiality was confirmed by means of transposon mutagenesis in 2003; and (3) the product of DdlA catalyzed reaction is d-ananly-d-alanine which promotes the cross-linking of PG by synthesizing the integrated pentapeptide. DdlA is responsible for supplying a substrate of d-alanyl-d-alanine for PG crosslinking.

d-cycloserine (DCS), a cyclic analogue of d-alanine and an effective second-line antibiotic against *Mtb*, inhibits the biosynthesis of the bacterial cell wall by targeting alanine racemase (Alr) and d-alanine:d-alanine ligase (Ddl). Nowadays, DCS is restricted to TB treatment due to its neurological side effects. DCS can be hydrolyzed to generate d-serine, which is a potent agonist at the glycine site of the NMDAR (*N*-methyl-d-aspartate-type glutamate receptor) which causes central nervous system side effects, such as headaches, drowsiness, depression, dizziness, vertigo, confusion, paresthesias, dysarthria, hyperirritability, psychosis, convulsions, and tremors. Overdose of DCS may result in paresis, seizures, and coma [6,14,15].

Nevertherless, *Tb*-DdlA is of particular importance as an effective and attractive anti-TB drug target. To obtain a better understanding of the DdlA profiles and develop a colorimetric assay for high-throughput inhibitor screening, we focused on explicating and characterizing *Tb*-DdlA which serves as a non-toxic and effective anti-TB compound screening.

## 2. Results

### 2.1. The Detection of Soluble Tb-DdlA Protein Expressed in Escherichia coli

**Figure 2:**
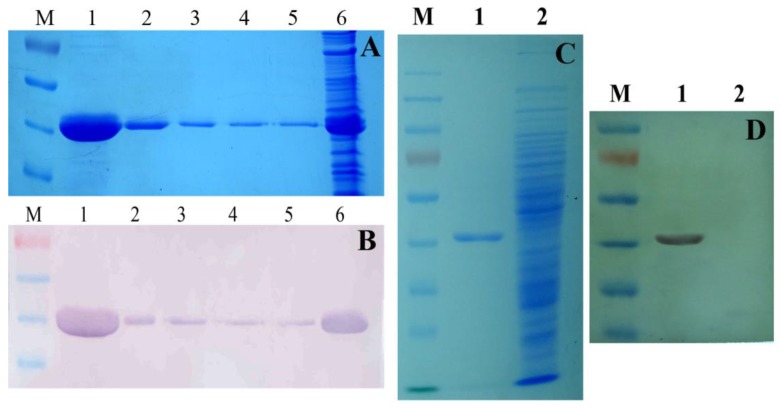
Analysis of the purified *Tb*-DdlA fusion protein by PAGE and Western blot. M, PageRuler prestained protein ladder (Fermentas), the sizes of the bands from top to bottom were 180 kDa, 130 kDa, 100 kDa, 70 kDa (red), 55 kDa, 40 kDa, 35 kDa, 25 kDa, and 15 kDa, respectively. (**A**) PAGE analysis of *Tb*-DdlA; 1–5, the elute fraction of *Tb*-DdlA fusion protein (with histidine tag) with an expected molecular weight of around 40 kDa purified by Ni^2+^-NTA affinity chromatography; 6, cell lysate from *E. coli* BL21(DE3) harboring pCold-*ddlA*; (**B**) Western Blot analysis of *Tb*-DdlA. The monoclonal anti-polyhistidine antibody at 1 to 5000 dilution was used to blot the expressed *Tb*-DdlA. The bands on the gel were visualized by BCIP/NBT solution; 1–5, the elute fraction of the *Tb*-DdlA fusion protein (with the histidine tag) with an expected molecular weight of around 40 kDa purified by Ni^2+^-NTA affinity chromatography; 6, cell lysate from *E. coli* BL21(DE3) harboring pCold-*ddlA*; (**C**) PAGE analysis of purified *Tb*-DdlA; 1, purified *Tb*-DdlA; 2, cell lysate from *E. coli* BL21(DE3) harboring pColdII; (**D**) Western Blot analysis of *Tb*-DdlA. The monoclonal anti-polyhistidine antibody at a 1 to 5000 dilution was used to blot the expressed *Tb*-DdlA. The bands on the gel were visualized by BCIP/NBT solution; 1, purified *Tb*-DdlA; 2, cell lysate from *E. coli* BL21(DE3) harboring pColdII.

The *Tb-ddlA* gene was cloned into the HindIII and NdeI sites of pColdII generating a *Tb*-DdlA fusion protein with an N-terminal histidine tag (His-tag), which serves as a selective and efficient tool for protein purification and detection. The expected molecular weight of the *Tb*-DdlA fusion protein was about 39.68 kDa of *Tb*-DdlA protein, plus 1 kDa of the His-tag. The expressed protein was purified by Ni^2+^-NTA affinity chromatography from supernatant of cell lysates, and was examined using polyacrylamide gel electrophoresis (PAGE) and Western blot. The PAGE and Western blot results displayed that only one band was visible on the gel and nitrocellulose membrane for the purified *Tb*-DdlA fusion protein, which had a molecular weight around 40 kDa, which was consistent with the expected size of *Tb*-DdlA fusion protein (about 40 kDa) (Figure 2A,B). Figure 2C,D shows that the monoclonal anti-polyhistidine antibody, which was used to blot the expressed *Tb*-DdlA, was specific for the expressed *Tb*-DdlA fusion protein with His-tag compared to its negative control. Therefore, the PAGE and Western blot analysis demonstrated that a soluble *Tb*-DdlA protein was successfully and highly-efficiently expressed as a fusion protein with histidine tag in *E. coli* BL21(DE3).

### 2.2. The Profiles of Tb-DdlA d-Alanyl-d-alanine Ligase Activity

#### 2.2.1. Confirmation of DdlA Activity

**Figure 3:**
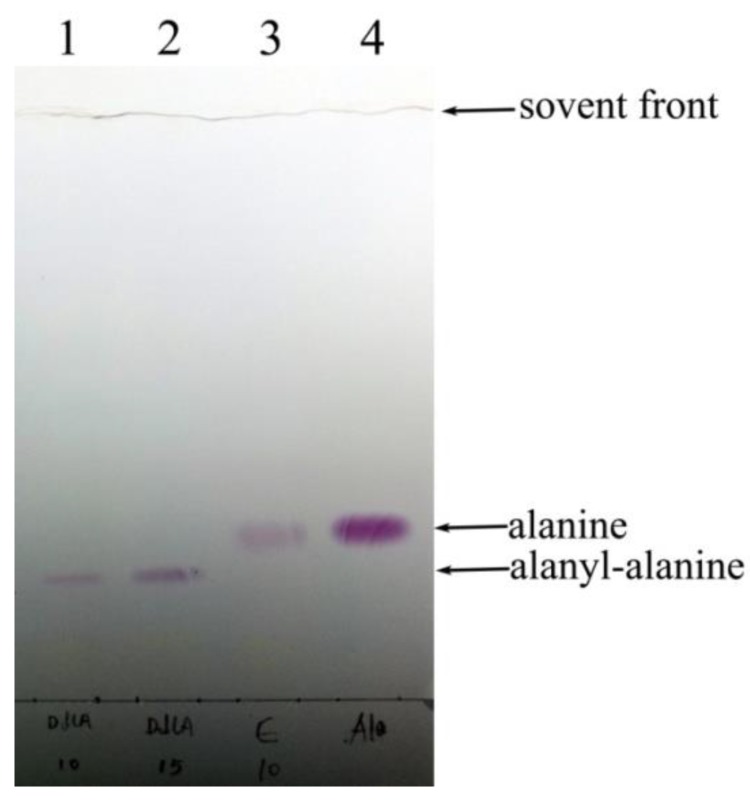
The identification of d-alanyl-d-alanine ligase activity of *Tb*-DdlA by HP-TLC. Phenol:water of 4:1 was used as the developing reagent. The color spots were visualized by spraying ninhydrin; **4**, the standard d-alanine; **3**, control, no DdlA in the reaction mixture; **2**, reactant, d-alanine was incubated with DdlA protein purified by Ni^2+^-NTA affinity choromatography; 15 μL was loaded; **1**, reactant, d-alanine was incubated with DdlA protein purified by Ni^2+^-NTA affinity choromatography; 10 μL was loaded.

The DdlA that catalyzes the dimerization of two d-alanine molecules typically couples ATP hydrolysis to provide a thermodynamic driving force and exhibits a cleavage of ATP to ADP and orthophosphate. Thus, in this study, the colorimetric assay demonstrated d-alanyl-d-alanine ligase activity of DdlA as evidence from orthophosphate released, which was measured by adding malachite green reagent. Table 1 showed a high d-alanyl-d-alanine ligase activity of DdlA. Thus, the coupled colorimetric assay demonstrated that *Tb*-DdlA encoded by *Rv2981c* possessed ligase activity toward d-alanine by liberating a molecule of orthophosphate. In addition, the d-alanyl-d-alanine ligase activity was confirmed by detecting the yielded d-Alanyl-d-alanine using HP-TLC. The HP-TLC was performed in the solvent system of phenol:water (4:1), and the color spots were visualized by spraying 2% ninhydrin solution. The HP-TLC image displays the band of d-alanyl-d-alanine formed by dimerization of two molecules of d-alanine (Figure 3). The d-alanine was completely converted into d-alanyl-d-alanine, so no remaining d-alanine was detected on the plate. The reaction mixture containing all reactants, except *Tb*-DdlA, was used as a negative control in this study. Therefore, the coupled colorimetric assay and HP-TLC image confirmed that *Tb*-DdlA encoded by *Rv2981c* possessed ligase activity toward d-alanine to generate a dipeptide of d-alanyl-d-alanine with liberation of a molecule of orthophosphate.

In addition, the Z-factor, a measure of the statistical effect size which has been proposed for use in high-throughput screening, was estimated according to the following equation: Z-factor = 1 − 3(σ_p_ + σ_n_)/│μ_p_ − μ_n_│ (parameters: μ, means; σ, standard deviation; p, positive of sample; n, negative control of sample). The Table 1 showed that the Z-factor of Reaction 2 was 0.735, which ranged between 0.5 and 1, so it was more reliable for high-throughput screening compared to Reaction 1, whose Z-factor was 0.187.

#### 2.2.2. Kinetic Analysis of *Tb*-DdlA

**Figure 4:**
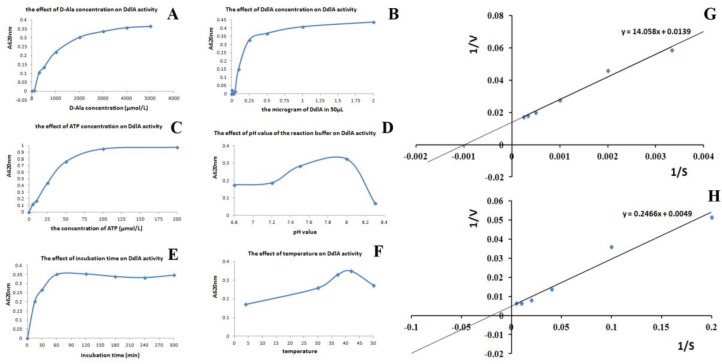
Determination of optimal reaction conditions for *Mtb* DdlA activity assay. (**A**–**F**) represented the effect of alanine concentration, DdlA concentration, ATP concentration, pH, incubation time, and temperature on the enzyme activity respectively; (**G**) The effect of alanine concentration on *Tb*-DdlA activity analyzed by the double reciprocal plot; (**H**) The effect of ATP concentration on *Tb*-DdlA activity analyzed by the double reciprocal plot.

The kinetic parameters of *Tb*-DdlA were measured using a malachite green reagent-coupled assay. The catalytic activity of *Tb*-DdlA was measured by varying d-alanine concentration, DdlA concentration, ATP concentration, and pH value in the reaction mixture, respectively, and incubation time and temperature, as well. *Tb*-DdlA was observed to display a higher d-alanyl-d-alanine ligase activity at more than 1 mmol/L of alanine concentration, more than 0.6 μg/mL of DdlA concentration, 50 μmol/L of ATP concentration, pH 8.0, and cultivating at 37 °C for more than 30 min (Figure 4). Therefore, an optimal enzymatic activity was determined according to the kinetic parameters and Z-factor as follows: 2 mmol/L alanine, 10 μg/mL DdlA, 75 μmol/L of ATP, and cultivating at 37 °C in the pH 8.0 reaction mixture for 60 min. In addition, the K_m_, V_max_, and K_cat_ values of the *Tb*-DdlA for the substrate of D-alanine and ATP were determined by a double-reciprocal plot under the optimal catalytic conditions. The K_mATP_ was 50.327 ± 4.652 μmol/L and the V_mATP_ was 204.082 ± 1.021 mmol/min/mg at pH 8.0 and 37 °C (Figure 4H); K_mAla_ was 1.011 ± 0.094 mmol/L and V_mAla_ was 71.942 ± 0.884 mmol/min/mg under the above optimal conditions of enzyme-catalyzed chemical reaction (Figure 4H). K_cat_ was 0.044 ± 0.002 min^−1^. Therefore, the double-reciprocal plot indicated that the enzyme exhibited a higher affinity to ATP than d-alanine.

#### 2.2.3. Inhibitory Analysis of *Tb*-DdlA Using DCS

**Figure 5:**
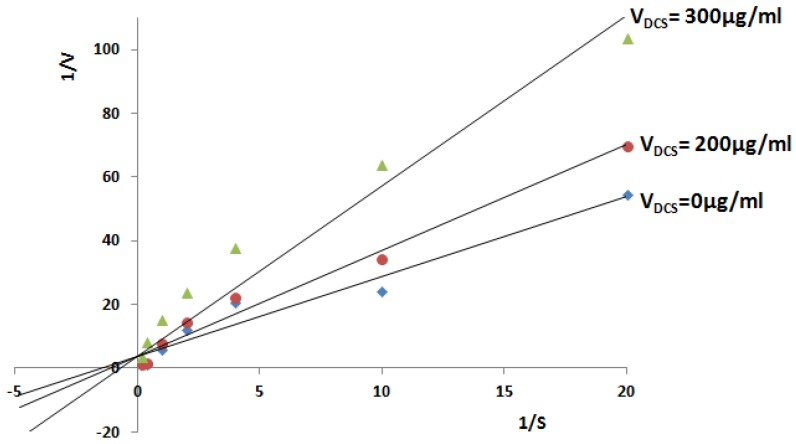
Steady state inhibition by DCS on d-alanyl-d-alanine ligase activity of DdlA. The double-reciprocal plots were obtained without or with different concentrations of DCS. The Lineweaver-Burk plot analysis of the data also confirmed the competitive inhibition.

To demonstrate whether the colorimetric assay to measure the released orthophosphate using malachite green reagent can be utilized for screening of *Tb*-DdlA’s inhibitory compounds, the DCS, a known competitive inhibitor typically with close structural similarities to d-alanine, was used as a positive control in this study. Double-reciprocal plots for the mode of *Tb*-DdlA inhibition by DCS with respect to the substrate of d-alanine were demonstrated herein. The concentrations of inhibitors including 0, 25, 50, 100, 200, 300 μg/mL were used in this study. However, only a high concentration of DCS (≥200 μg/mL (1.959 mmol/L)) exhibited an obviously inhibitory effect on *Tb*-DdlA (Figure 5), and the inhibitory effect enhanced as the concentration of DCS increased.

### 2.3. The Identification of *Tb*-DdlA Potential Interaction Partners

**Figure 6:**
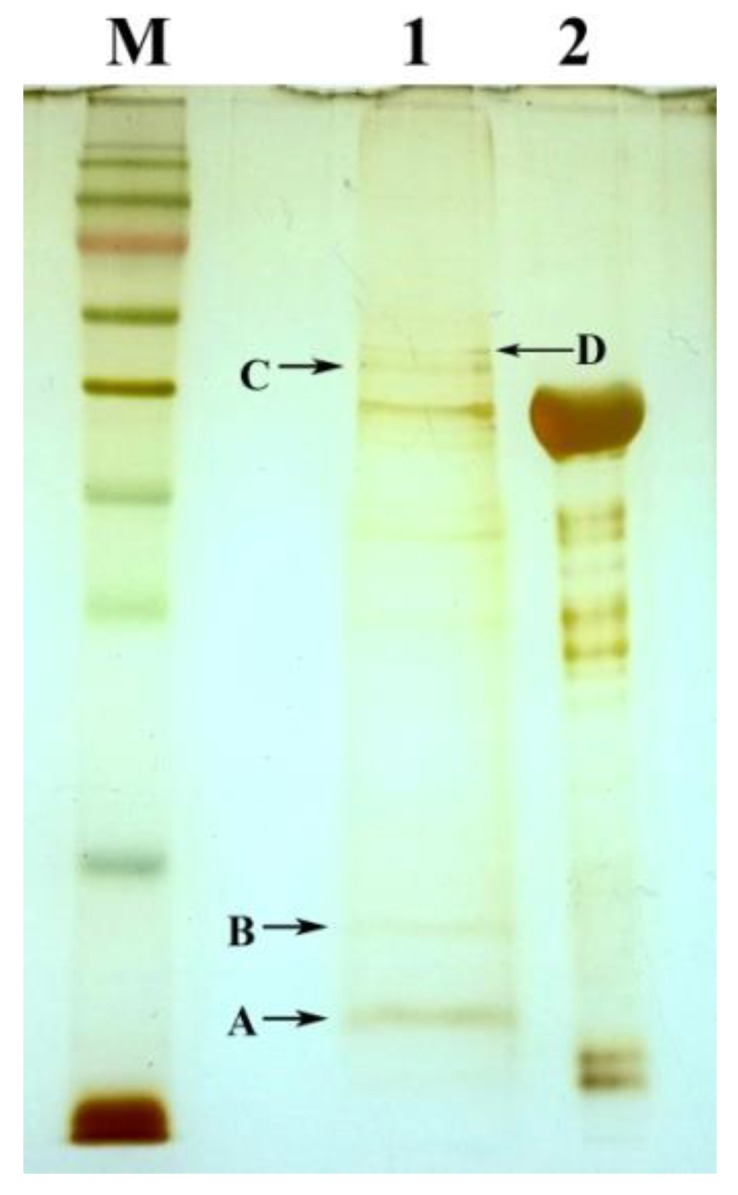
The PAGE analysis on *Tb*-DdlA interaction partners obtained by pull-down assay. The gel was stained by silver staining. **M**, PageRuler prestained protein ladder (Fermentas), the band sizes from top to bottom are 180 kDa, 130 kDa, 100 kDa, 70 kDa (red), 55 kDa, 40 kDa, 35 kDa, 25 kDa, 15 kDa, and 10 kDa, respectively; **1**, *Tb*-DdlA interaction protein complexes obtained by pull-down assay; **2**, *Tb*-DdlA purified by Ni^2+^-NTA affinity chromatography. **A**–**D**, the bands selected for MS identification.

Identifying interaction partners is key to understanding the function of a protein. In this study, the pull-down assay was used to obtain proteins in *Msm* that complexed with *Tb*-DdlA using *Tb*-DdlA-coated magnetic beads. To establish the pull-down assay, we expressed *Tb*-DdlA as a recombinant His_6_-fusion protein in *E. coli* and purified it to homogeneity. The loaded *Tb*-DdlA fusion protein was covalently coupled to Ni^2+^-NTA-activated sepharose beads, which were used to pull down putative interacting proteins from cell lysates of *Msm*. After washing, the putative interacting proteins were eluted and analyzed by PAGE and silver staining. In total, four extra bands were obtained by pull-down techniques (Figure 6).

The proteins from four bands on PAGE were deciphered as putative interaction partners of *Tb*-DdlA by mass spectrometry. The MS/MS analysis resulted in identification of eight different potential interacting partners of *Tb*-DdlA in total (Table 2). The list contained two well-known interactors which were involved in protein expression, including LuxR family transcriptional regulator (cupin) and elongation factor Tu (Tuf). The remaining five putative interacting partners were FAD-dependent oxidoreductase, ornithine-oxo-acid transaminase, diaminopimelate decarboxylase, cyclopropane-fatty-acyl-phospholipid synthase, and carbon-monoxide dehydrogenase large subunit, respectively. The last one was the hypothetical protein LI98_12890.

## 3. Discussion

The DdlA, an essential gene product determined by Himar1-based transposon mutagenesis in H37Rv strain [16], can dimerize two d-alanine molecules to generate the dipeptide of d-alanyl-d-alanine, which can be utilized as a substrate for PG biosynthesis and maturation. *Tb*-DdlA is of particular importance as an effective and attractive anti-TB drug target. In this study, we successfully expressed and purified soluble *Tb*-DdlA and analyzed its enzymatic characteristics. In addition, according to the kinetic parameters of the enzyme, we developed a colorimetric assay to measure the released orthophosphate for high-throughput screening of *Tb*-DdlA inhibitory compounds. During enzyme activity test, some disadvantages were found: (1) the color compound formed by malachite green reagent and orthophosphate had low solubility, so that an insoluble precipate was generated in the reaction mixture when it was catalyzed by *Tb*-DdlA with high activity. According to this property, we optimized the enzymatic parameters and Z-factor as follows: 2 mmol/L alanine, 10 μg/mL DdlA, 75 μmol/L of ATP in the pH 8.0 reaction mixture at 37 °C for 60 min. (2) malachite green reagent easily turned green in color even without adding anything, so we needed to perform color developing carefully in case the malachite green reagent was contaminated. Most importantly, the colorimetric assay to measure the released orthophosphate allowed a fast and accurate determination of *Tb*-DdlA’s ligase activity. Actually, in order to overcome the disadvantage of enzyme activty determination by adding malachite green reagent, we introduced a colorimetric assay of *Tb*-DdlA activity to monitor the decreased d-alanine using d-amino acid oxidase mixture (phenol, 4-aminoantipyrine, d-amino acid oxidase, peroxidase). However, the enzyme assay to detect the decreased d-alanine performed far lower sensitivity than to detect the released orthophosphate by adding malachite green reagent.

Usually, for inhibitory compound screening, the persistent enzyme activity was expected. However, during the enzyme activity assay, we found that *Tb*-DdlA only could maintain its high activity in 3 days after purification. On the second day, *Tb*-DdlA remained high activity, the absorbance reached to 0.758 ± 0.038 at 620 nm; then the enzyme’s activity started to decline and the absorbance of the assay was read as 0.452 ± 0.099 at 620 nm on the third day; until the fourth day, *Tb*-DdlA lost almost all of its activity (A_620nm_ was about 0.124 ± 0.002). For inhibitory compound screening of *Tb*-DdlA, it was required to prepare the fresh enzyme or improve the storage conditions for *Tb*-DdlA. DCS definitely exhibited an inhibitory effect on mycobacteria. Thus, we had perform the DCS inhibitory effect on *Msm* cells using various final concentrations of DCS containing 0, 5, 10, 25, 50, 100, and 200 μg/mL. The results showed that 50 μg/mL (0.490 mmol/L) of DCS inhibited the cell growth of *Msm* efficiently in vivo. In this study, the double reciprocal plot indicated that high concentrations of DCS (≥200 μg/mL) displayed inhibitory effects on *Tb*-DdlA. DCS, a structural analogues of d-alanine, competitively inhibit the bacterial growth by blocking the activity of DdlA and alanine racemase (Alr), theoretically [17,18], and its inhibitory effect depends on the concentration of DCS and d-alanine. In this study, the optimal concentration of d-alanine was determined as 2 mmol/L, so it required high concentrations of DCS (200 μg/mL (1.959 mmol/L); 300 μg/mL (2.939 mmol/L)) to inhibit *Tb*-DdlA activity, while less than 100 μg/mL (0.979 mmol/L) did not display any inhibitory effect on *Tb*-DdlA. The double-reciprocal plot also showed that *Tb*-DdlA exhibited a higher affinity to ATP (K_mATP_: 50.327 ± 0.465 μmol/L) than alanine (K_mAla_: 1.011 ± 0.094 mmol/L).

The study of protein–protein interactions (PPIs) exhibits crucial roles in the field of medicine, pharmaceutical industry, and biology. Identifying protein interaction sites and uncovering the interaction mechanism is closely relevant in the drug development industry. In addition, unveiling of protein interaction partners allows biologists to construct protein interaction networks, which in turn facilitate the understanding of many biological and clinical observations [19,20,21,22]. The data in this study indicated that *Tb*-DdlA could not maintain its activity long in vitro under the conditions tested, which might be attributable to its synergistic effect with potential interacting proteins that stabilized its activity in vivo. Therefore, we performed a pull-down assay to identify the interaction partners of *Tb*-DdlA in *Msm*. Thus, a *Tb*-DdlA fusion protein with His-tag was expressed for detecting potential interaction partners of *Tb*-DdlA in *Msm* using Ni^2+^-NTA magnetic agarose beads. In this study, *Msm* has been used as a surrogate model instead of pathogenic and slower-growing mycobacterial species such as *Mtb* [23]. *Msm* is a non-pathogenic and fast growing species with highly genetic and antigenic homology with *Mtb* [24]. *Msm* had similar unique cell wall to *Mtb*. Additionally, the homology between *Tb*-DdlA and its counterpart of *Sm*-DdlA in *Msm* had been analyzed. The alignment analysis showed that *Tb*-DdlA was highly homologous to *Sm*-DdlA (the identities accounted for 79%, and the positives accounted for 87%). Therefore, it was applicable for pull-down assay to be performed using cell lysates of *Msm* as a prey.

**Figure 7:**
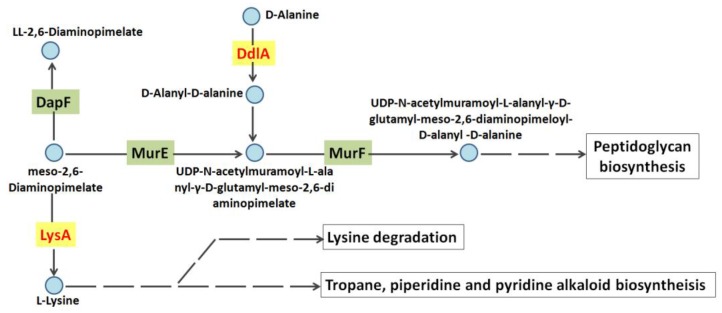
A view of metabolic pathway of DdlA and LysA leading to peptidoglycan biosynthesis. The pathway was summarized from the KEGG website (http://www.kegg.jp/kegg-bin/highlight_pathway?scale=1.0&map=mtu00300&keyword=lysA and http://www.kegg.jp/kegg-bin/highlight_pathway?scale=1.0&map=mtu01502&keyword=ddlA). Single lines represent the proceeding individual biosynthesis pathway catalyzed by the highlighted gene products. Dashed lines represent a series of successive biosynthesis pathways. Round nodes displayed metabolite intermediates. The genes associated with individual reaction were highlighted in grey color.

Totally, eight different potential interaction partners of *Tb*-DdlA had been identified in this study. Among these eight proteins, diaminopimelate decarboxylase (LysA) was of great interests. This enzyme was emphasized because it was particularly important with regard to PG biosynthesis. LysA participates in lysine biosynthesis, and catalyzes the conversion of lysine from meso-2,6-diaminoheptanedioate (DAP) with a liberation of CO_2_ molecule. DAP metabolic pathway has been attracting considerable attention due to its unique features in prokaryotes and its essentiality to cross-linking of PG which provides strength and rigidity to the cell wall in bacteria. It has been documented that mycobacterial cell walls contained an unusual high content of DAP [13,25,26,27,28]. The dipeptide d-alanyl-d-alanine, which catalyzed by DdlA was known to act as a substrate for the synthesis of petapeptides of PG as well. Figure 7 showed that DdlA and LysA had a close relationship in the pathway of PG synthesis. Therefore, we proposed a hypothesis that DdlA and LysA might act synergistically in PG biosynthesis in prokaryotes under the same regulation networks. The d-amino acids, such as DAP and d-alanine, play a crucial role in defining the cellular growth, cell wall integrity and protein synthesis of bacteria. In view of their importance, the designing of potential inhibitory compounds against any enzyme of the regulation networks might display a novel class of antitubercular agents which inhibit bacterial cell wall synthesis and protein biosynthesis, and are not toxic to mammalian species. Interestingly, LuxR family transcriptional regulator and elongation factor Tu were detected as interaction partners of *Tb*-DdlA, meanwhile, we performed the pull-down assay for the carboxypeptidase coded by Rv3627c, which hydrolyze the terminal d-alanine from the petapeptides of PG to promote PG crosslink in mycobacteria. The MS identification of Rv3627c interaction partners demonstrated that the above two proteins identified by *Tb*-DdlA were detected as interaction partners of Rv3627c, as well (data not published). We hypothesized that the LuxR family transcriptional regulator and elongation factor Tu were involved in the transcription and translation of *Tb*-DdlA and Rv3627c in mycobacteria, thus, it provided a promising view on anti-mycobacterial drug design that a compound which specifically targets LuxR family transcriptional regulator or elongation factor Tu of prokaryotes may be vital to bacterial survival.

In summary, we developed an easy and fast colorimetric assay for *Tb*-DdlA, which could be utilized for high-throughput screening of *Tb*-DdlA inhibitory compounds under the reaction conditions determined herein. In addition, we presented an analysis on *Tb*-DdlA interaction partners by the combination of pull-down assay with MS/MS. Eight putative interaction partners of *Tb*-DdlA had been identified in this study. Thus, our dataset provided a valuable resource for exploring *Tb*-DdlA biology.

## 4. Methods

### 4.1. Strains, Plasmids, and Growth Conditions

The characters of plasmids and bacterial strains used in this context were summarized in Table 3. *Escherichia coli* NovaBlue and BL21(DE3) cells were grown in Luria-Bertani (LB) broth or LB agar at 37 °C routinely. *Tb*-DdlA expression was performed at 16 °C. For the final concentration of antibiotic, 100 μg/mL ampicillin (Amp) was used in this study.

### 4.2. Construction of *Tb*-ddlA Expression Plasmids

The *Mtb ddlA* gene (*Rv2981c*) was amplified from *Mtb* H37Rv genomic DNA (supplied by Colorado State University through the NIH contract “Tuberculosis research materials and vaccine testing.”) using the forward primer of 5′ AG CATATG AGT GCT AAC GAC CGG CGT G 3′ (underlined sequence was NdeI site) and the reverse primer of AT AAGCTT CTA GTG CAG GCC CAC GCC GCG 3′ (underlined sequence was HindIII site). The purified PCR product was cloned into pMD18-T to generate pMD18-*Tb-ddlA* recombinant plasmid. Then the sequence-confirmed *ddlA* was cloned into pCold II, which carries a hexahistidine tag and a cold start promoter. The new derived recombinant plasmid was named as pCold II-*Tb-ddlA*, which was transformed into *Escherichia coli* BL21(DE3) for expression of the fusion protein.

### 4.3. Expression, Purification, and Identification of *Tb*-DdlA

For protein expression, *E. coli* BL21(DE3) carrying pCold II-*Tb ddlA* was grown in 200 mL LB broth containing Amp at 37 °C until the optical density reached 0.5 at 600 nm, followed by induction with 0.25 mmol/L isopropyl-d-thiogalactopyranoside (IPTG) at 16 °C for another 20 h. The cells were harvested and lysed by sonication (30s pulse with 40 s cooling interval) in chilled lysis buffer (20 mmol/L Tris-HCl, pH 8.0, 500 mmol/L NaCl, 20% glycerol, 1 mmol/L phenylmethylsulphonyl fluoride [PMSF]). Lysates were clarified by centrifugation at 27,000× *g* for 40 min, twice. The cytoplasmic fraction was applied to a pre-equilibrated Ni-NTA column (Qiagen) of 1.0 mL column volume. The column was washed with 20 mL wash buffer (lysis prep buffer with 20 mmol/L imidazole), and eluted with 10 mL elute buffer (lysis prep buffer with 200 mmol/L imidazole), 1 mL was collected for each tube, and the final protein concentration of the elute was determined by BCA kit.

The eluted fractions were analyzed by running a 12% SDS-polyacrylamide gel in a vertical electrophoresis apparatus and transferred to a nitrocellulose membrane in blotting buffer (20 mmol/L Tris-base, 150 mmol/L glycine and 20% methanol) to make the proteins accessible to antibody detection. The probing of the membrane with antibody of (anti)-polyhistidine monoclonal HIS-1 (Sigma, St. Louis, MO, USA) was conducted manually followed by incubation with the secondary antibody of antimouse-IgG conjugated alkaline phosphatase (Sigma, St. Louis, MO, USA), and the colorimetric detection of protein bands were developed by BCIP/NBT solution.

### 4.4. Functional Assay of *Tb*-DdlA

The enzyme’s activities were assayed in a 96-well microtiter plate at a total volume of 50 μL at 37 °C for 60 min. The reaction mixture was composed of 100 mmol/L HEPES (pH 8.0, 100 mmol/L KCl, 100 mmol/L MgCl_2_, 100 μmol/L ATP), and 2 mmol/L d-Ala. The purified *Tb*-DdlA (20 μg/mL or 10 μg/mL) was added to start the reaction after all assay components except the *Tb*-DdlA were pre-incubated at 37 °C for 10 min. The enzyme activities were monitored by measuring the release of orthophosphate. The concentration of released orthophosphate was measured by adding malachite green reagent (0.03% (*w*/*v*) malachite green, 0.2% (*w*/*v*) ammonium molybdate, and 0.05% (*v*/*v*) Triton X-100 in 0.7 N HCl) at 37 °C for 5 min. The plates were read at 620 nm by a microplate reader (Thermo Scientific Multiskan Ascent, Thermo Fisher Scientific Oy FI., Vantaa, Finland).

### 4.5. Kinetic Analysis of *Tb*-DdlA

The steady-state kinetic parameters were routinely evaluated by measuring the release of orthophosphate in microtiter plate. The optimized assay was performed in a final volume of 50 μL, and the absorbance was measured at 620 nm. The initial velocity was obtained by performing the enzymatic reaction at different incubation times and different concentrations of the purified enzyme. Then, kinetic parameters of *Tb*-DdlA catalyzed reaction were determined in the range of initial velocity. The optimum pH was determined using HEPES-NaOH at the pH of 7.2, 7.5, 8.0, and 8.3. The thermal stabilities of *Tb*-DdlA were determined by measuring enzyme activities at 4 °C, 30 °C, 37 °C, 42 °C, and 55 °C in the HEPES-NaOH buffer (pH 8.0). The Michaelis constant (K_m_) and maximal reaction velocity (V_m_) were determined by linear regression analysis based on the Lineweaver-Burk equation. The K_mD-Ala_ and V_mD-Ala_ against d-alanine was determined by carrying out the enzymatic reaction at various d-alanine concentrations including 0, 0.05, 0.1, 0.25, 0.5, 1, 2.5, and 5 mmol/L, and K_mATP_ and V_mATP_ against ATP was determined by performing the enzymatic reaction at various ATP concentrations containing 0, 5, 10, 25, 50, 100, 200, 400, 500, and 1000 μmol/L. In this study, each of enzymatic reactions was conducted in triplicate. To eliminate the interference of buffer components, the background absorbance of the reaction mixture without *Tb*-DdlA was measured and subtracted from the absorbance values with *Tb*-DdlA.

### 4.6. Colorimetric Inhibition Assay of *Tb*-DdlA by DCS

The antibiotic DCS, a known substrate competitive inhibitor of *Tb*-DdlA, was used to measure the inhibitory effects on *Tb*-DdlA protein. A double reciprocal plot was performed in the presence of inhibitor (0, 25, 50, 100, 200, and 300 μg/mL) under the following conditions using 100 mM HEPES (pH 8.0, 100 mmol/L KCl, 100 mmol/L MgCl2, 100 μmol/L ATP and a series of gradient concentrations of d-Ala including 0, 0.05, 0.1, 0.25, 0.5, 1, 2.5, 5 mmol/L), and 0.25 μg (5 μL) purified *Tb*-DdlA in a total volume of 50 μL at 37 °C for 60 min. DdlA activity was monitored by measuring the release of orthophosphate at 620 nm.

### 4.7. Pull down Assay of *Tb*-DdlA

Three microgram of purified His_6_-tagged *Tb*-DdlA (bait) was bound with Ni-NTA magnetic agarose beads suspension by rotating slowly at 4 °C for 1 h, then the supernatant was removed by using the magnetic MagRack6™ (Qiagen, Hong Kong) to leave only the magnetic beads with bound proteins in a microcentrifuge tube. The bound *Tb*-DdlA were incubated with 200 μL soluble proteins (prey) from the whole-cell lysates of *Msm* with gentle rotation at 4 °C for 1 h. The resins were washed to remove non-specific bound proteins. The bound proteins were eluted and electrophoresed by 12% SDS-PAGE. Gel bands were excised for a further MS/MS analysis after visualization by silver staining kit (Sigma, St. Louis, MO, USA). The detailed process of pull-down assay was performed as the manufacturer’s instructions.

### 4.8. MS/MS Analysis

Silver-stained gel slices were excised into small pieces followed by decolorization in 100 mmol/L NH_4_HCO_3_/30%ACN with K_3_Fe(CN)_6_ and Na_2_S_2_O_3_. Then, the digestion of decolorized gel was performed in 100 mmol/L NH_4_HCO_3_ solution containing 12.5 ng/μL of sequencing-grade trypsin for 20 h at 37 °C according to a modified in-gel trypsin digestion procedure. Mass spectrometry was carried out by using a 5800 MALDI-TOF/TOF MS (AB SCIEX, Foster City, CA, USA) and conducted in positive ionization mode and automatic data acquisition mode. The optimal source/gas parameters were set as follows: the instrument contained a Nd:YAG laser; the mass analyzer scan was 800–4000 Da *m/z*; the ions were accelerated with a voltage of 2 kV; the list of peptides of interest meant for the fragmentation analyses was manually defined in the equipment’s current settings with 2 kV collision energy and CID off; data were acquired using Mascot 2.2 software (Matrix Science, http://www.matrixscience.com). The obtained MS/MS spectra were searched against the NCBI database. 

## Figures and Tables

**Table 1:** The assay of d-alanyl-d-alanine ligase activity on *Tb*-DdlA. The decreasing d-alanine in the reaction mixture was measured by colorimetric reaction coupled with malachite green reagents. The analysis was conducted in quadruplicate.

Reactions	Components		Mean Values of A_620nm_ ± SD	Net Mean ± SD	Z-Factor
Reaction 1	100 μmol/L ATP; 2 mmol/L d-Ala	20 μg/mL DdlA	0.692 ± 0.099	0.452 ± 0.099	0.187
	100 μmol/L ATP; 2 mmol/L d-Ala	No DdlA	0.240 ± 0.023		
Reaction 2	75 μmol/L ATP; 2 mmol/L d-Ala	10 μg/mL DdlA	0.459 ± 0.013	0.291 ± 0.013	0.735
	75 μmol/L ATP; 2 mmol/L d-Ala	No DdlA	0.168 ± 0.013		

The above values in net mean ± SD had been deducted from values in DdlA group minus ones in its control group.

**Table 2:** The list of the putative interaction partners of *Tb*-DdlA in *Msm* detected by MS/MS. A, B, C and D were the corresponding bands from the silver-stained polyacrylamide gel.

ID	Protein Name	Accession No. (NCBI)	Locus Name/Gene Definition	Protein MW (D)	Protein PI	Pep. Count	Protein Score	Protein Score C. I. %
A	LuxR family transcriptional regulator	AIU17347.1	MSMEG_5707, cupin	12,011.3	6.36	5	285	100
B	hypothetical protein LI98_12890	AIU21016.1	MSMEG_2589	15,412.9	6.28	7	314	100
C	elongation factor Tu	YP_885786.1	MSMEG_1401, tuf	43,708.6	5.18	18	696	100
	FAD-dependent oxidoreductase	AIU20161.1	MSMEG_1682, FMO	46,185.2	5.86	13	74	99.697
	ornithine-oxo-acid transaminase	WP_011727654.1	MSMEG_1413, rocD	44,661.1	5.62	14	72	99.581
D	diaminopimelate decarboxylase	YP_889210.1	MSMEG_4958, lysA	50,317	5.25	13	224	100
	cyclopropane-fatty-acyl-phospholipid synthase	YP_890503.1	MSMEG_6284	48,608.7	5.99	13	198	100
	carbon-monoxide dehydrogenase large subunit	WP_011727162.1	MSMEG_0746	85,807	5.21	19	60	93.366

**Table 3:** Bacterial strains and plasmids used in this study.

Strains/Plasmids	Description	Source
Strains		
*E. coli* NovaBlue	Used for cloning and propagation of plasmids	Novagen
*E. coli* BL21(DE3)	Used for expressing *Tb*-DdlA protein	Invitrogen
Plasmids		
pMD18-T	Carries *ampR* gene; used for cloning PCR product with A at 3′ ends	Takara
pCold II	Carries *ampR* gene; contains cold start promoter; used for expressing *M. tuberculosis* DdlA	Takara
pMD18-*Tb*-*ddlA*	Carries *ampR* gene; *M. tuberculosis ddlA* was cloned to the EcoRV site of pMD18-T	This study
pCold II-*Tb*-*ddlA*	Carries *ampR* gene; used for expressing *M. tuberculosis* DdlA	This study
